# Supplementary material for: PSMA PET Imaging and Therapy in Adenoid Cystic Carcinoma and Other Salivary Gland Cancers: A Systematic Review
Source: Cancers (Basel). 2022 Jul 22;14(15):3585. doi: 10.3390/cancers14153585 (PMC9330626; doi:10.3390/cancers14153585)
Supplement: Supplementary file 1 [file cancers-14-03585-s001.zip › cancers-1750752-supplementary.pdf]

Table S1. Overall weighted mean SUVmax for each adenoid cystic carcinoma patient [23].

Patient	Subsite (no of Lesions)	Mean SUVmax	Overall Mean Weighted SUVmax	Weighted Mean SUVmax for Local Recurrence Only	Weighted Mean SUVmax for Metastases Only
1	Neck metastases (2)	9.15			
	Lung metastases (53)	9.12	9.12		9.12
2	Lung metastases (16)	7.76	7.76		7.76
3	Lung metastases (51)	9.98	9.98		9.98
4	Local recurrence (1)	13.80			
	Lung metastases (10)	6.98			
	Pleural metastasis (1)	6.6			
	Pericardial metastases (2)	10.5			
	Myocardial metastasis (1)	8.9			
	Subcutaneous metastases (6)	6.95			
	Bone metastases (3)	11.1	8.13	13.80	7.88
5	Lung metastases (18)	4.85	4.85		4.85
6	Bone metastases (6)	10.68	10.68		10.68
7	Local recurrence (1)	4.8			
	Neck metastasis (1)	2.5	3.65	4.8	2.5
8	Lung metastases (28)	5.29	5.29		5.29
9	Pleural metastases (29)	6.37			
	Bone metastasis (1)	5.9	6.35		6.35
10	Lung metastases (27)	14.9	14.9		14.9
11	Local recurrence (1)	10.6		10.6	
	Kidney metastasis (1)	11.1	10.85		11.1
12	Lung metastases (2)	7.8	7.8		7.8
13	Lung metastases (30)	6.7			
	Liver metastases (6)	10.6	7.35		7.35
14	Lung metastasis (1)	6.2			
	Bone metastases (4)	3.9	4.36		4.36
15	Lung metastases (43)	4.38	4.38		4.38
Range mean SUVmax		2.5-14.9	3.65 – 14.9	4.8 – 13.8	2.5 – 14.9
Total weighted mean SUVmax			115.02	29.2	114.3
Weighted mean SUVmax			7.67	9.73	7.62

Table S2. Overall weighted mean SUVmax for each adenoid cystic carcinoma patient [19].

Patient	Subsite (no of Lesions)	Mean SUVmax	Overall Mean Weighted SUVmax	Weighted Mean SUVmax for Local Recurrences Only	Weighted Mean SUVmax for Metastases Only	Tumour/Liver Ratio
1	Leptomeningeal	8.71				8.71/4.83 = 1.8
	Local recurrence	7.06	7.89	7.06	8.71	7.06/4.83 = 1.46
2	Lungs	0				-
	Peritoneal	0				-

	Liver	0				-
	Iliac crest	2.04	2.04		2.04	2.04/5.35 = 0.38
3	Lungs	2.66				2.66/2.33 = 1.14
	Liver	4.01	3.34		3.34	4.01/2.33 = 1.72
4	Intracranial	12.81				12.81/2.83 = 4.53
	Vertebra	3.47	8.14		8.14	3.47/2.83 = 1.23
5	Leptomeningeal	2.42				2.42/3.24 = 0.75
	Local recurrence	2.42	2.42	2.42	2.42	2.42/3.24 = 0.75
6	Lungs	3.64	3.64		3.64	3.64/2.74 = 1.33
7	Lungs	4.68				4.68/4.86 = 0.96
	Local recurrence	2.41	3.55	2.41	4.68	2.41/4.86 = 0.50
8	Local recurrence	2.62	2.62	2.62	-	2.62/3.82 = 0.69
9	Iliac crest	12.97				12.97/4.89 = 2.65
	Lungs	6.66	9.82		9.82	6.66/4.89 = 1.36
Range mean SUVmax		0-12.97	2.04 – 9.82	2.41 – 7.06	2.04 – 12.97	0.38 – 4.53
Total weighted mean SUVmax			43.46	14.51	42.79	
Weighted mean SUVmax			4.83	3.63	5.35	

Table S3. Overall weighted mean SUVmax for each adenoid cystic carcinoma patient [34].

Patient	Subsite (no of Lesions)	Mean SUVmax	Overall Mean Weighted SUVmax	Weighted Mean SUVmax for Local Recurrences Only	Weighted Mean SUVmax for Metastases Only
1	Lung	3.5	3.5		3.5
2	Intracranial	6.5	6.5		6.5
3	Pelvis	10.2	10.2		10.2
6	Parapharyngeal	7.0	7.0	7.0	
Range mean SUVmax		3.5 – 10.2	3.5 – 10.2	-	3.5-10.2
Total weighted mean SUVmax			27.2	7.0	20.2
Weighted mean SUVmax			6.8	7.0	6.73

Table S4. Overall weighted mean SUVmax for each adenoid cystic carcinoma patient (combined from Table S1, S2, S3 and findings from Konig et. al. (2017) [35]).

	Mean SUVmax	Overall Weighted Mean SUVmax	Weighted Mean SUVmax for Local Recurrences Only	Weighted Mean SUVmax for Metastases Only
Range mean SUVmax	2.04 – 23.35	2.04 – 23.35	2.41 – 13.8	2.04 – 14.9
Total weighted mean SUVmax		209.03	50.71	177.29
Weighted mean SUVmax		7.21	6.33	6.82

Table S5. Overall weighted mean SUVmax per metastatic subsite for adenoid cystic carcinoma [19, 23, 34, 35].

Subsite	Patient no (no of Lesions)	Mean SUVmax	Total Weighted Mean SUV max for Region
Lung	1 (53)	9.12	
	2 (16)	7.76	
	3 (51)	9.98	
	4 (10)	6.98	
	5 (18)	4.85	
	8 (28)	5.29	
	10 (27)	14.9	
	12 (2)	7.8	
	13 (30)	6.7	
	14 (1)	6.2	
	15 (43)	4.38	
	2 (x)	0	
	3 (1)	2.66	
	6 (1)	3.64	
	7 (1)	4.68	
	8 (1)	6.66	
	1 (1)	3.5	7.94 (2.66 – 14.9)
Neck	1 (2)	9.15	
	7 (1)	2.5	6.93 (2.5 – 9.15)
Cardiac	4 (2) Pericardium	10.5	
	4 (1) myocardium	8.9	9.97 (8.9 – 10.5)
Bone	4 (3)	11.1	
	6 (6)	10.68	
	9 (1)	5.9	
	14 (4)	3.9	
	2 (1)	2.04	
	4 (1)	3.47	
	9 (1)	12.97	
	3 (1)	10.2	8.20 (2.04 – 12.97)
Subcutaneous	4 (6)	6.95	6.95
Liver	13 (6)	10.6	
	3 (1)	4.01	8.45 (4.01 – 10.6)
Kidney	11 (1)	11.1	11.1
Leptomeningeal & intracranial	1 (1)	8.71	
	5 (1)	2.42	
	4 (1)	12.81	
	2 (1)	6.5	7.61 (2.42 – 12.81)
Right maxillary sinus	1 (1)	23.35	23.35

Table S6. Overall weighted mean SUVmax for other salivary gland cancer patients [23].

Patient	Subsite (no of Lesions)	Mean SUVmax	Overall Mean Weighted SUVmax	Weighted Mean SUVmax for Local Recurrence Only	Weighted Mean SUVmax for Metastases Only
16	Neck metastases (2)	5.05			
	Bone metastases (4)	3.45	3.98		3.98
17	Neck metastasis (1)	5.8			
	Lung metastases (5)	4.12			
	Lymph nodes (12)	5.50	5.13		5.13
18	Lymph node (1)	5.7			
	Local recurrence (1)	10.9			
	Bone metastasis (1)	10.2	8.93	10.9	7.95
19	Neck metastases (3)	1.43			
	Lung metastases (6)	1.78			
	Lymph nodes (3)	4.87	2.47		2.47
20	Local recurrence (1)	4.0	4.0	4.0	-
21	Brain metastases (13)	2.84			
	Local recurrence (1)	16.8			
	Bone metastases (23)	14.27	10.32	16.8	10.14
22	Lung metastases (28)	5.24			
	Lymph nodes (4)	4.73			
	Liver metastases (7)	8.81			
	Bone metastases (8)	5.14			
	Subcutaneous (2)	2.10	5.56		5.56
23	Bone metastases (22)	6.20	6.2		6.2
24	Lung metastases (13)	1.85	1.85		1.85
25	Neck metastases (7)	4.63			
	Lung metastasis (1)	4.5			
	Pericardial metastasis (1)	3.2			
	Lymph nodes (4)	3.83			
	Subcutaneous metastasis (1)	2.9	1.20		1.20
Range mean SUVmax		1.43-16.8	1.2-10.32	4.0 – 16.8	1.43-14.27
Total weighted mean SUVmax			49.64	31.7	44.48
Weighted mean SUVmax			4.96	10.57	4.94

Table S7. Overall weighted mean SUVmax for other salivary gland cancer patients [34].

Patient	Subsite (no of Lesions)	Mean SUVmax	Overall Mean Weighted SUVmax	Weighted Mean Suvmax For Local Recurrences Only	Weighted Mean SUVmax for Metastases Only
4	Pelvis (1)	12.5			12.5
5	Pelvis (1)	9.7			9.7

Range mean SUVmax	9.7 – 12.5	9.7 – 12.5	-	9.7 – 12.5
Total weighted mean SUVmax		22.2	-	22.2
Weighted mean SUVmax		11.1	-	11.1

Table S8. Overall weighted mean SUVmax for each other salivary gland cancer patient (combined from S6 and S7).

	Mean SUVmax	Overall Weighted Mean SUVmax	Weighted Mean SUVmax for Local Recurrences Only	Weighted Mean SUVmax for Metastases Only
Range mean SUVmax	1.43-16.8	1.2 – 12.5	4.0 – 16.8	1.43-14.27
Total weighted mean SUVmax		71.84	31.7	66.68
Weighted mean SUVmax		5.99	10.57	6.06

Table S9. Overall weighted mean SUVmax per metastatic subsite for other salivary gland cancer patients [23].

Subsite	Patient no (No of lesions)	Mean SUVmax	Total weighted mean SUV max for region
Lung	17 (5)	4.12	
	19 (6)	1.78	
	22 (28)	5.24	
	24 (13)	1.85	
	25 (1)	4.5	3.90 (1.78 – 5.24)
Lymph nodes	17 (12)	5.50	
	18 (1)	5.7	
	19 (3)	4.87	
	22 (4)	4.73	
	25 (4)	3.83	5.02 (3.83 – 5.7)
Neck	16 (2)	5.05	
	17 (1)	5.8	
	19 (3)	1.43	
	25 (7)	4.63	4.05 (1.43 – 5.8)
Cardiac	25 (1)	3.2	3.2
Bone	16 (4)	3.45	
	18 (1)	10.2	
	21 (23)	14.27	
	22 (8)	5.14	
	23 (22)	6.2	
	4 (1)	12.5	
	5 (1)	9.7	9.19 (3.45 – 14.27)
Subcutaneous	22 (2)	2.10	
	25 (1)	2.9	2.37 (2.1 – 2.9)
Liver	22 (7)	8.81	8.81
Intracranial	21 (13)	2.84	2.84
